# Neuroendocrine Tumors (NETs) of the Larynx †

**DOI:** 10.3390/diagnostics16193096

**Published:** 2026-09-24

**Authors:** Jan Laco, Abbas Agaimy, Alfio Ferlito

**Affiliations:** 1 The Fingerland Department of Pathology, University Hospital Hradec Kralove, 500 05 Hradec Kralove, Czech Republic; 2 The Fingerland Department of Pathology, Faculty of Medicine in Hradec Kralove, Charles University, 500 03 Hradec Kralove, Czech Republic; 3 Institute of Pathology, Erlangen University Hospital, Friedrich Alexander University of Erlangen-Nuremberg, 91054 Erlangen, Germany; abbas.agaimy@uk-erlangen.de; 4 Comprehensive Cancer Center, European Metropolitan Area Erlangen-Nuremberg (CCC ER-EMN), Friedrich Alexander University of Erlangen-Nuremberg, 91054 Erlangen, Germany; 5 International Head and Neck Scientific Group, 35100 Padua, Italy; profalfioferlito@gmail.com

**Keywords:** larynx, neuroendocrine neoplasms, neuroendocrine tumors, terminology, classification, diagnosis, prognosis

## Abstract

To review the current state of knowledge regarding neuroendocrine tumors (NETs) of the larynx, with a particular focus on their epidemiology, etiopathogenesis, clinico-pathological features, immunohistochemical profile, and differential diagnostics. Narrative review. Diagnostic criteria for laryngeal NETs were recently defined in the 5th edition of the WHO Classification of Head and Neck Tumors, which newly introduces the entity of NET, grade 3 (G3 NET). However, some of these criteria are still considered provisional. The most common NET of the larynx is the G2 NET. These tumors typically affect older men, with the supraglottic region being the most frequent localization. For a definitive diagnosis, it is essential to demonstrate the epithelial origin and neuroendocrine differentiation of these tumors using cytokeratins and neuroendocrine markers (e.g., chromogranin, INSM1, synaptophysin). The differentiation between individual NET types is based on mitotic activity and/or Ki-67 proliferation index, as well as the presence or absence of necrosis. Laryngeal NETs typically exhibit wild-type p53 expression and retained Rb1 and somatostatin receptor 2 expression. This can be utilized in the differential diagnosis from neuroendocrine carcinomas (NECs). Given that laryngeal NETs frequently express calcitonin, they must be differentiated from medullary thyroid carcinoma (MTC), particularly in lymph node metastases. A high frequency of *HRAS* gene mutations has been recently demonstrated in laryngeal NETs, underlining their distinctness from their gastroenteropancreatic and thoracopulmonary counterparts. By utilizing the proposed diagnostic criteria for individual types of laryngeal NETs, it is anticipated that more precise knowledge regarding the clinico-pathological characteristics of these exceedingly rare tumors will be obtained, particularly with respect to their biological behavior and prognosis.

## 1. Introduction

Analogous to their gastroenteropancreatic counterparts, differentiated neuroendocrine tumors (NETs) arising in the upper aerodigestive tract and salivary glands are defined in the current 5th edition of the WHO Classification of Head and Neck Tumours as “well-differentiated epithelial neuroendocrine neoplasms (NENs). In this volume, they are discussed in a separate chapter, together with neuroendocrine carcinomas (NECs) and paragangliomas [1].

Neuroendocrine neoplasms (NENs) of the larynx comprise a heterogeneous group of diseases whose classification has evolved significantly over the past 50 years, driven by advancements in understanding their molecular genetic alterations and biological behavior [2,3,4]. This review summarizes the current state of knowledge regarding this group of tumors, based on the information provided in the 5th edition of the WHO Classification of Head and Neck Tumours and three previous review articles [1,2,3,4]. Additionally, recent cases of these tumors published between 2020 and 2026, usually as case reports or smaller series, are discussed [5,6,7,8,9,10,11,12,13,14,15,16,17,18,19].

Although the three previous reviews have addressed NENs of the larynx [2,3,4], none incorporated the diagnostic framework introduced by the 5th edition of the WHO Classification of Head and Neck Tumours 2024, which for the first time defines NET grade 3 as a distinct entity with still-provisional criteria [1]. Given the extreme rarity of these tumors, the persistent heterogeneity of historical terminology across successive classification schemes, and the accrual of 46 additional cases published between 2020 and 2026, an updated synthesis was considered necessary to consolidate current diagnostic criteria and to provide an aggregate analysis of recently reported cases that, to our knowledge, has not been previously performed.

## 2. Materials and Methods

This review article involved a search of the MEDLINE/PubMed, Scopus, and Google Scholar electronic databases using the following search terms: “larynx”, “neuroendocrine neoplasm”, “neuroendocrine tumor”, and “neuroendocrine carcinoma”. All included articles were required to be written in English. The titles and abstracts were initially screened by the first author (J.L.) to exclude irrelevant publications. Publications were considered eligible when they provided clinically, pathologically, molecularly, or therapeutically relevant information on laryngeal epithelial NENs. Publications focused exclusively on NECs, paragangliomas, non-laryngeal primary tumors, or unrelated neuroendocrine neoplasms were excluded unless they contained information directly relevant to the classification or differential diagnosis of laryngeal NETs. Subsequently, the full texts were reviewed by all authors, who also checked the reference lists to identify any additional relevant publications not captured by the initial electronic search. The literature search was last updated in July 2026, and articles published after this date were not systematically captured. Given the extreme rarity of laryngeal NETs, the marked heterogeneity of diagnostic terminology across historical classification systems, and the small, heterogeneously treated case series reported in the literature, a formal systematic review or meta-analysis with pooled quantitative estimates was not considered methodologically appropriate and was not attempted, as combining such heterogeneous data could generate a spurious impression of precision. Because the review addresses multiple complementary diagnostic, pathological, and prognostic questions rather than a single focused comparative clinical question, a formal PICO framework was not considered applicable to the scope of this narrative review. Instead, this narrative review was structured following the quality criteria proposed by the Scale for the Assessment of Narrative Review Articles (SANRA) [20], including an explicit strategy, transparent screening criteria, and independent full-text review by all authors. As this manuscript was designed as a narrative review rather than a systematic review, no review protocol was prospectively registered in PROSPERO or another systematic-review registry. Of the 96 records identified through the electronic search, 81 were excluded after title/abstract screening (duplicates, wrong anatomic site, diagnosis other than NET, or absence of confirmed neuroendocrine differentiation), yielding the 15 references [5,6,7,8,9,10,11,12,13,14,15,16,17,18,19] describing individual cases or small series published between 2020 and 2026.

## 3. Historical Perspectives and Current Classification

The first description of a well-differentiated neuroendocrine carcinoma of the larynx was likely published in 1969 by Goldmann et al., who reported a typical carcinoid involving the laryngeal surface of the epiglottis and the anterior commissure in a 73-year-old man [21]. Another report followed in 1980, when Markel et al. described a laryngeal (typical) carcinoid [22]; however, a subsequent review suggested it was more likely an atypical carcinoid [23]. In the same year, Gehanno et al. described another case of laryngeal carcinoid, albeit without detailed histological documentation [24]. Consequently, two cases published by Duvall et al. in 1983 are often considered the first well-documented report of a laryngeal carcinoid [25].

A landmark study by Wenig et al., published in 1988, introduced a new classification system for laryngeal neuroendocrine tumors, inspired by the contemporary classification for pulmonary NECs [26]. The authors proposed categorizing laryngeal NECs into well-differentiated (synonymous with carcinoid), moderately differentiated (synonymous with atypical carcinoid), and poorly differentiated carcinomas (synonymous with small cell carcinoma). Notably, this classification omitted large cell NEC (LCNEC), as well-established diagnostic criteria for this malignancy had not yet been established, even in pulmonary pathology. The first description of a laryngeal LCNEC was subsequently published by Greene et al. in 2005 [27].

The gradual evolution of laryngeal NENs classification is well illustrated by the shifting terminology across successive editions of the WHO Classification of Head and Neck Tumours, as summarized in Table 1. In the 3rd and 4th editions, the description of laryngeal NENs was integrated directly into the chapter on laryngeal tumors. Conversely, the current 5th edition discusses NENs in a dedicated chapter, together with head and neck paragangliomas, ectopic or invasive PitNETs/pituitary adenomas, and Merkel cell carcinoma.

**Table 1:** Comparison of laryngeal epithelial NENs classification across the last three editions of the WHO Classification of Head and Neck Tumours [1,28,29].

3rd Edition WHO	4th Edition WHO	5th Edition WHO
Typical carcinoid	Well-differentiated neuroendocrine carcinoma	Neuroendocrine tumor, grade 1
Atypical carcinoid	Moderately differentiated neuroendocrine carcinoma	Neuroendocrine tumor, grade 2
---	---	Neuroendocrine tumor, grade 3
Small cell carcinoma, neuroendocrine type	Small cell neuroendocrine carcinoma	Small cell neuroendocrine carcinoma
---	Large cell neuroendocrine carcinoma	Large cell neuroendocrine carcinoma
Combined small cell carcinoma, neuroendocrine type, with non-small cell carcinoma	Combined small cell neuroendocrine carcinoma Combined large cell neuroendocrine carcinoma	Combined small cell carcinoma *

Abbreviations: NEN = neuroendocrine neoplasm. * This category corresponds to the MiNEN category in the gastroenteropancreatic tract. The non-NEC component in head and neck tumors can be SCC (mostly) or any other rare subtype (e.g., intestinal-type adenocarcinoma in the sinonasal tract).

The 3rd edition, published in 2005, divided laryngeal NENs into five categories: typical carcinoid, atypical carcinoid, small cell carcinoma of neuroendocrine type, combined small cell carcinoma of neuroendocrine type with non-small cell carcinoma, and paraganglioma [28]. It was noted that some atypical carcinoids may fulfill the diagnostic criteria for LCNEC of the lung. The subsequent 4th edition, published in 2017, revised this schema into well-differentiated, moderately differentiated, and poorly differentiated NECs, with the latter further subdivided into small cell and large cell NECs (SCNEC, LCNEC) [29].

In the current 5th edition, published in printed version in 2024, NENs are covered in a distinct Chapter 15, entitled “Neuroendocrine neoplasms and paraganglioma”. The current terminology classifies epithelial NENs of the upper aerodigestive tract and salivary glands into well-differentiated NENs called NETs—further subclassified into NET grade 1 (G1 NET), NET grade 2 (G2 NET), and NET grade 3 (G3 NET)—while poorly differentiated NENs called SCNEC (including combined SCNEC) and LCNEC are addressed separately [1]. The proposed diagnostic criteria for these tumors are shown in Table 2.

**Table 2:** Diagnostic criteria for epithelial NENs of the upper aerodigestive tract and salivary glands according to the 5th edition of the WHO Classification of Head and Neck Tumours [1].

Neuroendocrine Neoplasm (NEN)	Tumor Type	Diagnostic Criteria
Well-differentiated NEN (NET)	NET, grade 1	No necrosis and <2 mitoses/2 mm^2^ Ki-67 < 20% ^a^
	NET, grade 2	Necrosis and/or 2–10 mitoses/2 mm^2^ Ki-67 < 20% ^a^
	NET, grade 3 ^a^	>10 mitoses/2 mm^2^ Ki-67 > 20% ^a^ Absence of NEC morphology
Poorly differentiated NEN (NEC)	Small cell NEC	>10 mitoses/2 mm^2^ Ki-67 > 20% (often >70%) Small cell NEC morphology
	Large cell NEC	>10 mitoses/2 mm^2^ Ki-67 > 20% (often >50%) Large cell NEC morphology

Abbreviations: NEC = neuroendocrine carcinoma; NEN = neuroendocrine neoplasm; NET = neuroendocrine tumor. ^a^ Provisional criteria applied at this time.

## 4. Epidemiology and Incidence

Due to the extreme rarity of well-differentiated NETs of the larynx, reliably determining their true incidence remains highly challenging, if not impossible [30,31]. This situation is further complicated by the fact that the precise diagnostic criteria for individual subtypes of these tumors were only defined relatively recently. It is highly probable that some cases diagnosed as atypical carcinoids (now G2 NETs) in the past actually represented LCNECs. Misdiagnosis of G2 NET as paraganglioma may have also occurred and resulted in a myth that the laryngeal paraganglioma has a high metastatic potential [3].

Laryngeal epithelial NENs are very rare, comprising much less than 1% of all laryngeal neoplasms [8]. But they still represent the second most common malignancy of the larynx, following squamous cell carcinoma (SCC) and accounting for 37% of all laryngeal non-SCC malignant tumors [32]. Overall, SCNEC is the most frequent laryngeal epithelial NEN, followed by G2 NET. The true incidence of G3 NET remains unknown, as only a few cases have been published to date. Remarkably, G3 NET was the most frequent type of laryngeal epithelial NEN diagnosed over the 10-year period in the study by Yue J et al. [14]. For context, bone/cartilage sarcomas constitute the third most common malignancy of the larynx [32].

The current WHO Classification of Head and Neck Tumours states that laryngeal NETs are more prevalent in men during their sixth and seventh decades of life, with a male-to-female ratio of 2.4:1 [1]. Selected clinico-pathological characteristics of 46 new cases of laryngeal NETs published between 2020 and 2026 are summarized in Table 3 [5,6,7,8,9,10,11,12,13,14,15,16,17,18,19]. For more detailed characteristics regarding treatment modalities, the reader is referred to the individual articles. Although the number of cases is small, a preliminary analysis of the data in Table 3 reveals that males predominate over females among these newly published laryngeal NETs as well, with a ratio reaching as high as 4.8:1. The median age falls between 56 and 67 years, with an overall range of 44 to 87 years. To our knowledge, this represents the first descriptive synthesis of clinico-pathological characteristics reported in laryngeal NET cases published since 2020.

**Table 3:** Selected clinico-pathological data of laryngeal NETs published in the period 2020–2026.

Reference No.	Diagnosis	Sex/ Age (Years)	Smoking	Site	Stage	Outcome/ Follow-Up (Months)
[5]	G2 NET	M/79	Ex-smoker	Epiglottis	I	NED/36
[6]	G2 NET	M/59	Smoker	Supraglottis	IVA	AWD/11
[7]	G2 NET	M/67	Smoker	Epiglottis	I	DOC/106
[8] *	G2 NET (9/9)	7M + 2F 56 (44–64)	Smokers 7/9	Supraglotis (9/9)	III–IVC	NED (2/8) ** AWD (4/8) DOD (2/8) 8 (6–74)
[9]	G2 NET	M/65	Smoker	Epiglottis	I	NED/18
[10]	G1 NET	M/67	Ex-smoker	Arytenoid	I	NED/48
[11]	G2 NET	M/80	NA	Arytenoid	I	DOD/120
[12]	G2 NET	M/74	Smoker	Epiglottis	I	DOD/51
[13]	G2 NET	M/67	Ex-smoker	Aryepiglottic fold	I	NED/12
[14]	G2 NET	F/72	Non-smoker	Epiglottis	I	NED/115
[14]	G2 NET	M/61	Smoker	Epiglottis	I	DOD/57
[14]	G1 NET	F/77	Smoker	Aryepiglottic fold	I	DOD/60
[14]	G3 NET	M/61	Smoker	Epiglottis	I	NED/57
[14]	G3 NET	M/65	Smoker	Epiglottis	IVA	DOD/16
[14]	G3 NET	M/75	Smoker	Aryepiglottic fold	I	NED/19
[14]	G3 NET	M/69	Smoker	Aryepiglottic fold	IVA	NED/10
[14]	G3 NET	M/68	Smoker	Epiglottis	IVA	AWD/10
[15]	G2 NET	F/71	Non-smoker	Epiglottis	IVB	DOD/15
[16]	G3 NET	M/76	Smoker	Supraglottis	IVA	NED/3
[17]	G2 NET	M/67	NA	NA	NA	AWD/18
[17]	G3 NET	M/87	NA	NA	NA	DOD/30
[18]	G2 NET	M/64	Smoker	Epiglottis	III	NED/15
[18]	G2 NET	M/67	Smoker	Epiglottis	III	NED/75
[18]	G2 NET	M/69	Smoker	Aryepiglottic fold	II	NED/37
[18]	G2 NET	M/54	Smoker	Epiglottis	III	DOD/67
[18]	G2 NET	F/60	Non-smoker	Arytenoid	I	AWD/18
[18]	G2 NET	M/70	Smoker	Arytenoid	I	AWD/6
[18]	G2 NET	F/60	Non-smoker	Arytenoid	I	NED/6
[18]	G2 NET	M/62	Smoker	Vocal cord	III	LFF/13
[19] *	7 G2 NET 2 G1 NET	8M + 1F (NA)	Smokers 6/9	Supraglottis 8/9 Glottis/Subglottis 1/9	I–II 6/9 III–IV 3/9	7/9 Alive *** 2/9 DOD 38 (10–164)

Abbreviations: AWD = alive with disease; DOC = death of other cause; DOD = death of disease; LFF = loss for follow-up; NA = not available; NED = no evidence of disease; NET = neuroendocrine tumor * Data for individual patients not provided in the article. ** One patient lost for follow-up. *** Unspecified if NED or AWD.

## 5. Etiopathogenesis

Currently, no definitive etiological factors have been identified for laryngeal NETs, with the potential exception of smoking, which may represent a risk factor predominantly for NECs and G2 NETs [1]. Remarkably, the vast majority of patients shown in Table 3 were smokers or ex-smokers. The role of high-risk human papillomavirus infection in the pathogenesis of laryngeal epithelial NENs has not been demonstrated [1]. Notably, they have not been reported as part of neuroendocrine tumor syndromes [1].

It is widely accepted that laryngeal NETs, much like laryngeal NECs, originate from neuroendocrine cells resident within the mucosal epithelium. However, a major caveat of this theory is that laryngeal NETs most frequently arise in the supraglottic region, specifically the epiglottis, whereas neuroendocrine cells are most densely populated within the mucosal epithelium of the subglottis [33]. An alternative theory suggests that these tumors develop from laryngeal epithelial progenitor cells that undergo malignant transformation driven by undetermined environmental factors, subsequently differentiating toward a neuroendocrine phenotype [34]. At present, it also remains unclear whether laryngeal NETs can increase their mitotic and proliferative activity—and consequently their grade—over the course of the disease or in response to treatment. Specifically, it is unknown whether yet unreported progression from a laryngeal G1 NET to a G2/G3 NET can occur, a phenomenon well-documented in NETs of the gastroenteropancreatic tract, which may contribute to treatment resistance [35].

## 6. Localization

In the head and neck area, NETs are most frequently localized in the sinonasal tract and the larynx. Within the larynx, they predominantly arise in the supraglottic region, particularly on the laryngeal surface of the epiglottis, the aryepiglottic fold, and the arytenoid cartilage [2,3]. Notably, only two patients shown in Table 3 presented with a tumor localized to the glottis/subglottis.

## 7. Clinical Features

The clinical symptoms of laryngeal NETs stem from their most common primary localization within the supraglottic region. These predominantly include dysphagia, odynophagia, a foreign body sensation, sore throat, hoarseness, an irritative cough, dyspnea, and hemoptysis. However, a subset of these tumors may remain asymptomatic, being discovered incidentally during laryngoscopy or intubation indicated for other reasons. If the regional lymph nodes are involved by metastases, this manifests as a palpable cervical mass [1].

Elevated calcitonin levels may rarely occur due to ectopic calcitonin secretion by the laryngeal NETs, necessitating differentiation from medullary thyroid carcinoma. In such scenarios, functional imaging studies (e.g., 68Ga-DOTA PET-CT) are valuable in distinguishing a laryngeal origin from a thyroid primary [6]. Very rarely, laryngeal NETs may present with carcinoid syndrome due to excessive hormone production [12,36].

## 8. Macroscopic Appearance and Microscopy

Macroscopically, laryngeal NETs typically appear as a polypoid, nodular, or exophytic mass, often measuring less than 20 mm.

Laryngeal NETs share the principal morphological features of well-differentiated NETs from other anatomical sites. They typically grow in the submucosa, and ulceration may occur. By definition, they are well-differentiated and composed of small nests, trabeculae, ribbons, or sheets of cells with uniform round to oval nuclei, dispersed (“salt-and-pepper”) nuclear chromatin and granular, pale acidophilic cytoplasm, often without distinct cell borders. Cells with clear, oncocytic, plasmacytoid or rhabdoid features may also occur. The stroma can be highly vascularized or hyalinized. G1 NETs exhibit fewer than 2 mitoses/2 mm^2^ and lack necrosis. G2 NETs demonstrate 2–10 mitoses/2 mm^2^ and/or exhibit necrosis. In both G1 and G2 NETs, the Ki-67 proliferation index is generally below 20%. Figure 1 and Figure 2 illustrate the microscopic findings of G1 and G2 NETs (Figure 1 and Figure 2). Although the formal diagnostic criteria for laryngeal G3 NETs remain to be fully established, current proposals include more than 10 mitoses/2 mm^2^, a Ki-67 proliferation index exceeding 20%, and the absence of NEC morphology [1]. In cases with discordance between mitotic count and Ki-67 proliferation index, the final grade is determined by the parameter indicating a higher, more aggressive grade. Laryngeal mixed neuroendocrine-non-neuroendocrine neoplasms (MiNENs) harboring a NET component are exceptional, with one report of combined G2 NET and SCC [37]. Importantly, a true MiNEN, which requires each histologically and immunohistochemically distinct component to account for at least 30% of the tumor population, must be distinguished from SCC with focal expression of neuroendocrine markers. In the latter, the tumor retains a monophasic squamous morphology without the true biphasic architectural diversity characteristic of MiNEN.

## 9. Immunohistochemistry and Molecular Genetics

Immunohistochemically, laryngeal NETs express cytokeratins and neuroendocrine markers (e.g., chromogranin A, INSM1, and synaptophysin) [1]. The use of cytokeratins (e.g., AE1/AE3, CAM5.2) is of particular importance, as their positivity distinguishes epithelial NETs from paragangliomas and from other NEN mimics, which typically lack expression of these markers. Routine use of Ki-67 staining is encouraged, as it offers a diagnostic advantage in small biopsies with crush artifacts that preclude morphological assessment and the counting of mitotic figures. Figure 1 and Figure 2 illustrate the immunohistochemical findings of G1 and G2 NETs (Figure 1 and Figure 2).

The Ki-67 proliferation index not only enables reliable classification of individual NET subtypes but also, in conjunction with p53 and Rb1 markers, helps differentiate them from NECs, in which the Ki-67 index is typically greater than 50%. While NECs, particularly SCNEC, exhibit a mutation-type of p53 expression and a loss of nuclear Rb1 expression, NETs are characterized by wild-type p53 expression and preserved nuclear Rb1 expression [38]. Although the distinction between NETs and NECs is usually H&E-based, these surrogate markers are valuable in ambiguous cases and in limited or crushed biopsies. Based on limited evidence, somatostatin receptor 2 (SSTR2) status can also aid in this differential diagnosis, as this marker is usually expressed in NETs, unlike in NECs, although the expression may be heterogeneous, particularly in G3 NETs [39]. Importantly, only membranous staining is considered specific. Heterogeneous SSTR2 expression—both intratumoral and intertumoral—remains a major diagnostic challenge; the latter involves different expression profiles between the primary tumor and, e.g., its lymph node metastasis. Of note, the immunohistochemical expression of SSTR2 is not equivalent to clinically relevant receptor expression on functional imaging, where a subset of NETs may have limited uptake and some NECs may retain expression.

It should be emphasized that expression of a single neuroendocrine marker is not sufficient to diagnose a NET, as this diagnosis should integrate compatible morphology, epithelial differentiation demonstrated by expression of cytokeratins, convincing expression of neuroendocrine markers, and exclusion of relevant mimics. The minimum immunohistochemical panel should include cytokeratins (e.g., AE1/AE3, CAM5.2), chromogranin A, INSM1, synaptophysin, and Ki-67.

A significant subset of laryngeal G2 NETs express calcitonin; consequently, nodal metastases from an occult laryngeal primary may be misdiagnosed as metastatic medullary thyroid carcinoma (MTC). Unlike most MTCs, patients with calcitonin-positive laryngeal NETs lack elevated serum calcitonin levels, although exceptions may occur [6]. Strong and diffuse expression of TTF-1 and monoclonal CEA, as well as the presence of somatic *RET* mutations, favors MTC over laryngeal NET [38].

Little is known about the molecular genetics of laryngeal NETs. Wild-type p53 expression and retained Rb1 expression can be interpreted as indirect evidence of the absence of *TP53* and *RB1* gene mutations, which are otherwise characteristic of NECs. In a recent comprehensive molecular profiling study of 15 laryngeal epithelial NENs, all four NETs (three G2 NETs and one G3 NET) harbored *HRAS* Q61L mutations, whereas these mutations were absent in all analyzed NECs [40]. It should be noted that this finding has so far been reported only as a conference abstract; full peer-reviewed data are awaited to confirm this observation. Given that *HRAS* mutations are uncommon in NETs arising in other anatomical sites, this finding may suggest a unique, organ-specific pathogenesis.

## 10. Differential Diagnostics

Accurate diagnosis of laryngeal NETs requires both confirmation of their epithelial origin using cytokeratins and verification of their neuroendocrine differentiation using immunohistochemical markers, with chromogranin A, INSM1, and synaptophysin being highly recommended [1]. Their differential workup aims at (1) confirming their epithelial and true neuroendocrine nature, (2) distinction from NECs, and (3) distinction from the many epithelial and mesenchymal mimics that may express neuroendocrine markers such as adamantinoma-like Ewing sarcoma and diverse other entities [41].

The differential diagnosis of these neoplasms primarily includes NECs, which must be distinguished particularly from G3 NETs. In addition to assessing the Ki-67 proliferation index, the immunohistochemical evaluation of p53, Rb1, and SSTR expression can be utilized. NETs typically exhibit wild-type p53 expression and preserved expression of both Rb1 and SSTR. Conversely, NECs, particularly SCNEC, are characterized by a mutation-type of p53 expression and a loss of both Rb1 and SSTR2 expression [38]. It must be once again emphasized that mitotic count above 10 per 2 mm^2^ and Ki-67 proliferation index above 20%, by themselves, are not sufficient to establish the diagnosis of G3 NET, as Ki-67 values may overlap between G3 NET and NEC. The diagnosis of NET requires unequivocally well-differentiated morphology and absence of SCNEC or LCNEC morphology. The proposed diagnostic algorithm is shown in Figure 3. Another critical differential diagnostic consideration is paraganglioma. In this scenario, cytokeratin staining is highly valuable, as paragangliomas are typically cytokeratin-negative [38]. The distinction from metastatic MTC in a lymph node has been described above.

## 11. Prognosis and Treatment

Evaluating the prognosis of laryngeal NETs with certainty remains highly challenging, primarily because their precise diagnostic criteria were established relatively recently [1]. G1 NETs are exceedingly rare, and the number of published cases is insufficient to reliably determine their prognosis. Although G2 NETs are substantially more common, it is widely assumed that a subset of tumors previously diagnosed as G2 NETs (formerly atypical carcinoids) actually represent cases of LCNEC. G3 NETs represent a newly defined tumor entity in the larynx; their diagnostic criteria remain provisional, and only a few isolated cases have been reported in the literature (see Table 3). Notably, Table 3 includes one case of a G1 NET where the patient died 60 months after diagnosis. Moreover, seven out of 28 patients (25%) in the G2 NET group died, while 2 out of 7 patients died in the G3 NET cohort.

Additional real-world evidence supporting this survival divergence comes from a recently accepted 15-year single-institution series by Qiao et al. [19]. They reviewed 31 consecutive patients with primary laryngeal epithelial NENs treated between 2010 and 2024. Well-differentiated NETs accounted for 29% of cases (7 G2 NETs, 2 G1 NETs; no G3 NET was identified), predominantly arising in the supraglottis (89%) and managed with curative-intent, function-preserving surgery. Patients with NET achieved markedly better outcomes than those with NEC, with 5-year overall survival of 44.4% versus 0% and 5-year disease-free survival of 50.0% versus 0% (both *p* < 0.001). On multivariate analysis, NEC subtype remained an independent predictor of both overall survival (HR 4.23) and disease-free survival (HR 3.67), outweighing T and N stage. Notably, the authors themselves acknowledged that, with only two G1 NET cases and no G3 NET represented, their cohort—like most published series—remains underpowered to characterize the prognosis at the extremes of the NET grading spectrum, reinforcing the point raised above.

Due to the rarity of laryngeal NETs, there are currently no universally accepted treatment guidelines. Most of these tumors are treated with surgical resection alone or a combination of surgery and adjuvant radiotherapy or platinum-based chemoradiotherapy (commonly platinum plus etoposide) [8]. However, the necessary extent of surgery remains unclear (organ-preserving approach vs. laryngectomy). Particularly in the case of G1 NETs and/or a cN0 status, it is unknown whether elective neck dissection is appropriate. The role of immunotherapy in the management of laryngeal NETs remains poorly defined. Specifically, further research is needed to determine whether agents such as nivolumab can demonstrate therapeutic efficacy comparable to that observed in laryngeal NECs, where partial responses have been documented in a subset of patients [42].

Regardless of grade, all NETs are malignant and require very long-term, or even lifelong, follow-up. This is because locoregional and distant metastases can occur a significant time after the removal of the primary tumor. In addition to assessing the locoregional status, follow-up evaluations must include a comprehensive examination of the patient, as distant metastases can develop in virtually any organ—most commonly the lungs, liver, and bones—as well as atypical sites such as the skin, breast, or peritoneum [8].

## 12. Limitations

As with any narrative review, the present article is subject to the inherent limitations of a non-systematic methodology, including the absence of a formal risk-of-bias assessment and a residual susceptibility to selection bias in the literature cited. However, given the current infeasibility of a methodologically sound systematic review or meta-analysis in this ultra-rare and terminologically heterogeneous tumor group, as discussed above, a structured narrative approach following SANRA criteria [20] was judged the most appropriate strategy currently available. Beyond the aggregate synthesis presented in Table 3, no new patient-level data are reported.

## 13. Conclusions and Future Directions

As evidenced by the detailed review of the current state of knowledge regarding NETs of the larynx, significant progress has been achieved in this field over recent decades. However, it is only recently that diagnostic criteria for these tumors have been defined. Although currently somewhat provisional, these criteria were inspired by the diagnostic frameworks of NENs in other organ systems, reflecting a broader effort to harmonize NEN classification across the entire spectrum of human pathology. Nevertheless, numerous questions regarding these neoplasms remain unresolved.

For instance, the issue of precursor lesions in the larynx remains poorly understood. It is entirely unclear whether the general principle—where neuroendocrine hyperplasia precedes the development of a NET—applies also to the larynx. Furthermore, comprehensive studies on the expression of novel immunohistochemical biomarkers (e.g., DLL3, ODF1, ATRX, menin) in these tumors are currently lacking, contrasting with the extensive research conducted on NETs in other anatomical sites. Similarly, the detailed molecular genetic characteristics of laryngeal NETs remain largely uncharacterized.

Substantial advancements in this field will likely only be achieved through multicenter studies capable of enrolling cohorts of dozens of patients presenting with these extremely rare malignancies. A review of the literature indicates that even major tertiary centers typically harbor no more than 20 cases of laryngeal NENs in their archives. Within the framework of these multicenter collaborative studies, it will be possible not only to validate the currently proposed, partly provisional diagnostic criteria for laryngeal NETs, but also to more precisely delineate the biological behavior of individual tumor subtypes. Moreover, such studies may facilitate the identification and characterization of additional cases of MiNENs with NET component, which remain exceptionally rare.

## Figures and Tables

**Figure 1:**
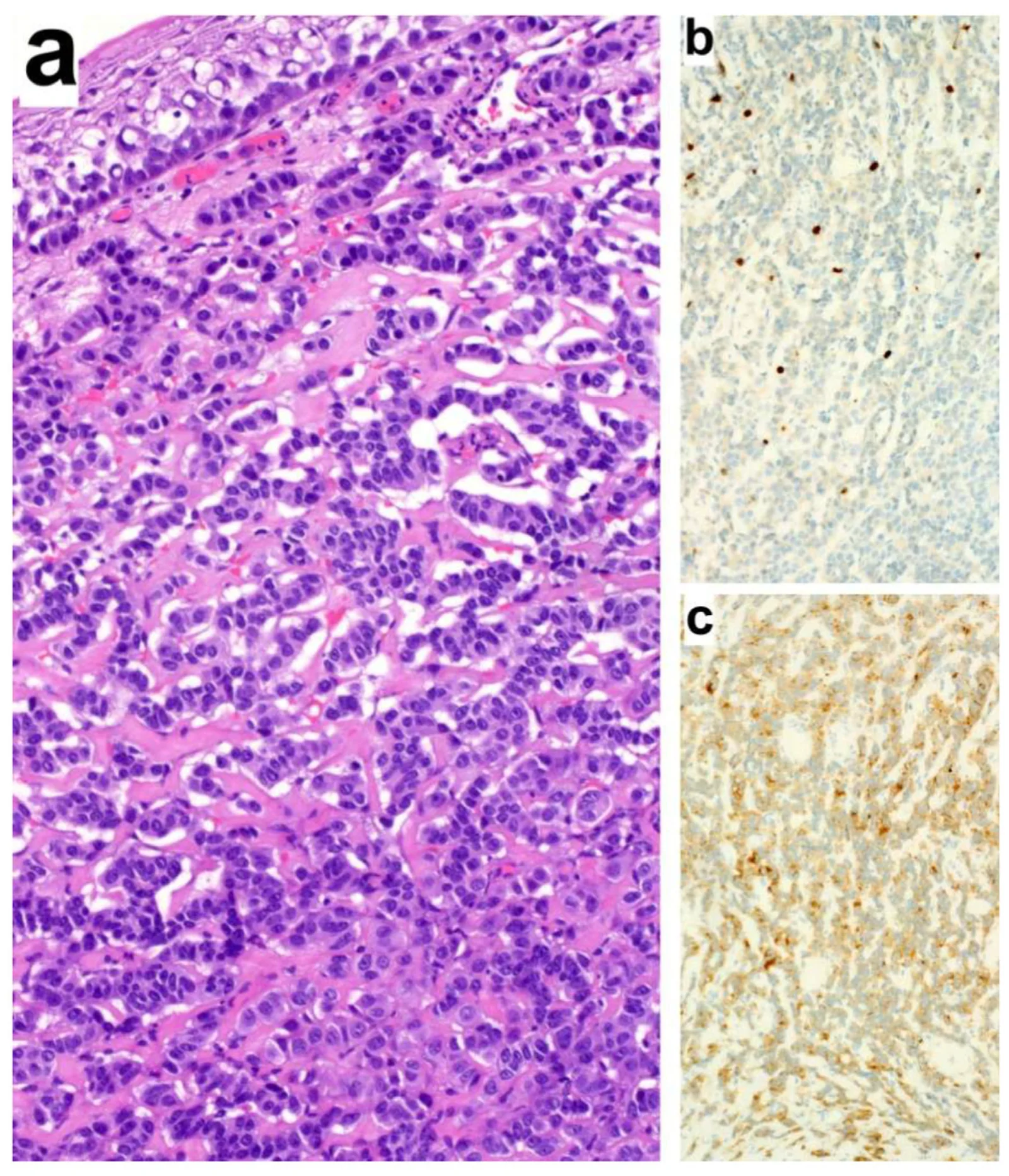
Grade 1 Neuroendocrine tumor (G1 NET). (**a**) The well-differentiated submucosal tumor is composed of trabeculae of monomorphic cells without mitotic activity (hematoxylin-eosin, original magnification 200×). (**b**) Proliferation index Ki-67 is <1% (immunohistochemistry, original magnification 200×). (**c**) Diffuse expression of synaptophysin (immunohistochemistry, original magnification 200×).

**Figure 2:**
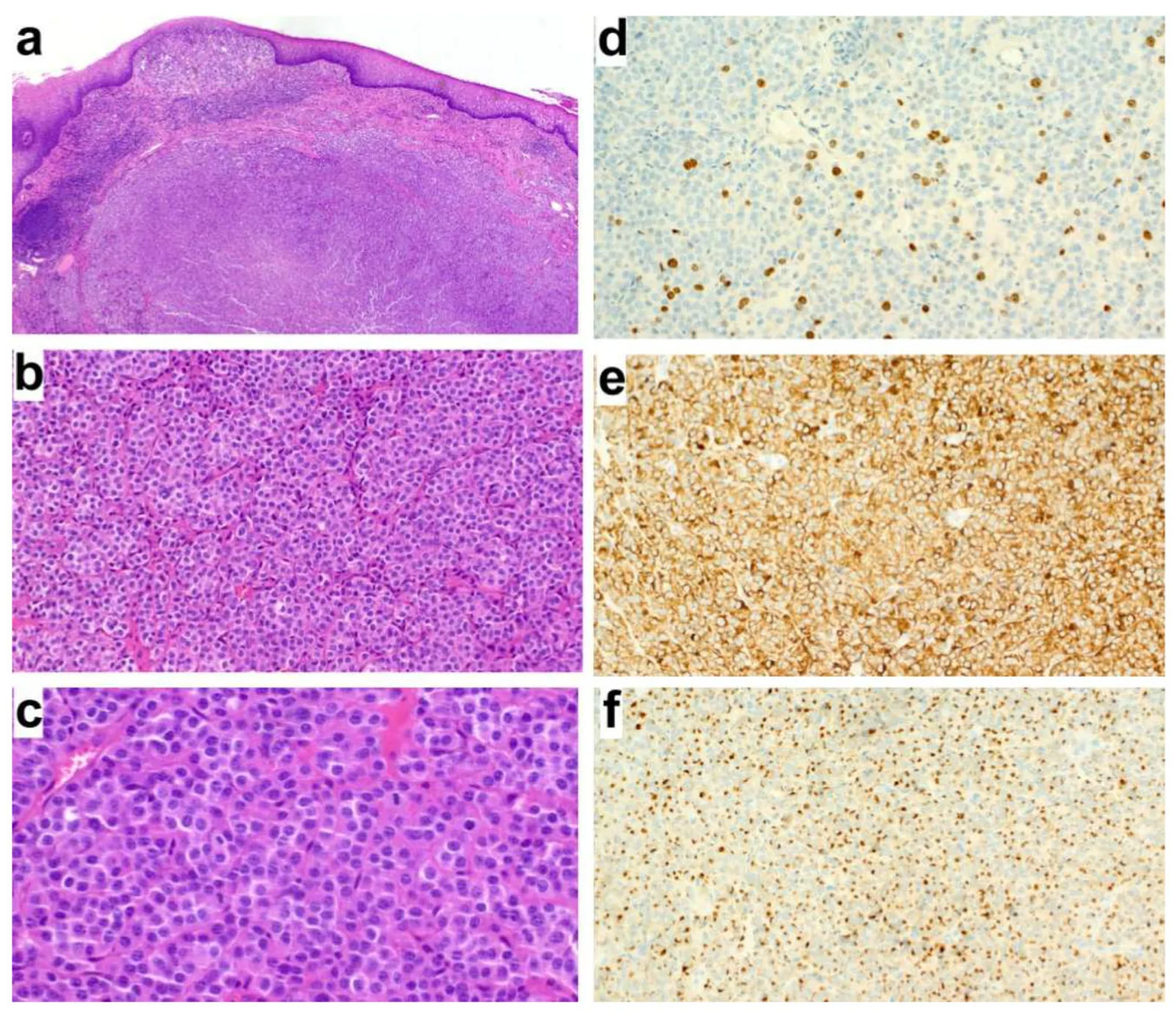
Grade 2 Neuroendocrine tumor (G2 NET). (**a**) The well-differentiated submucosal tumor grows beneath the mucosal squamous epithelium (hematoxylin-eosin, original magnification 40×). (**b**) The tumor is composed of nests of monomorphic cells (hematoxylin-eosin, original magnification 200×). (**c**) Three mitotic figures are visible in this field, with a total mitotic count of 7 mitoses per 2 mm^2^ (hematoxylin-eosin, original magnification 400×). (**d**) Proliferation index Ki-67 is 6% (immunohistochemistry, original magnification 200×). (**e**) Diffuse expression of cytokeratins (AE1/AE3) (immunohistochemistry, original magnification 200×). (**f**) Diffuse expression of chromogranin A (immunohistochemistry, original magnification 200×).

**Figure 3:**
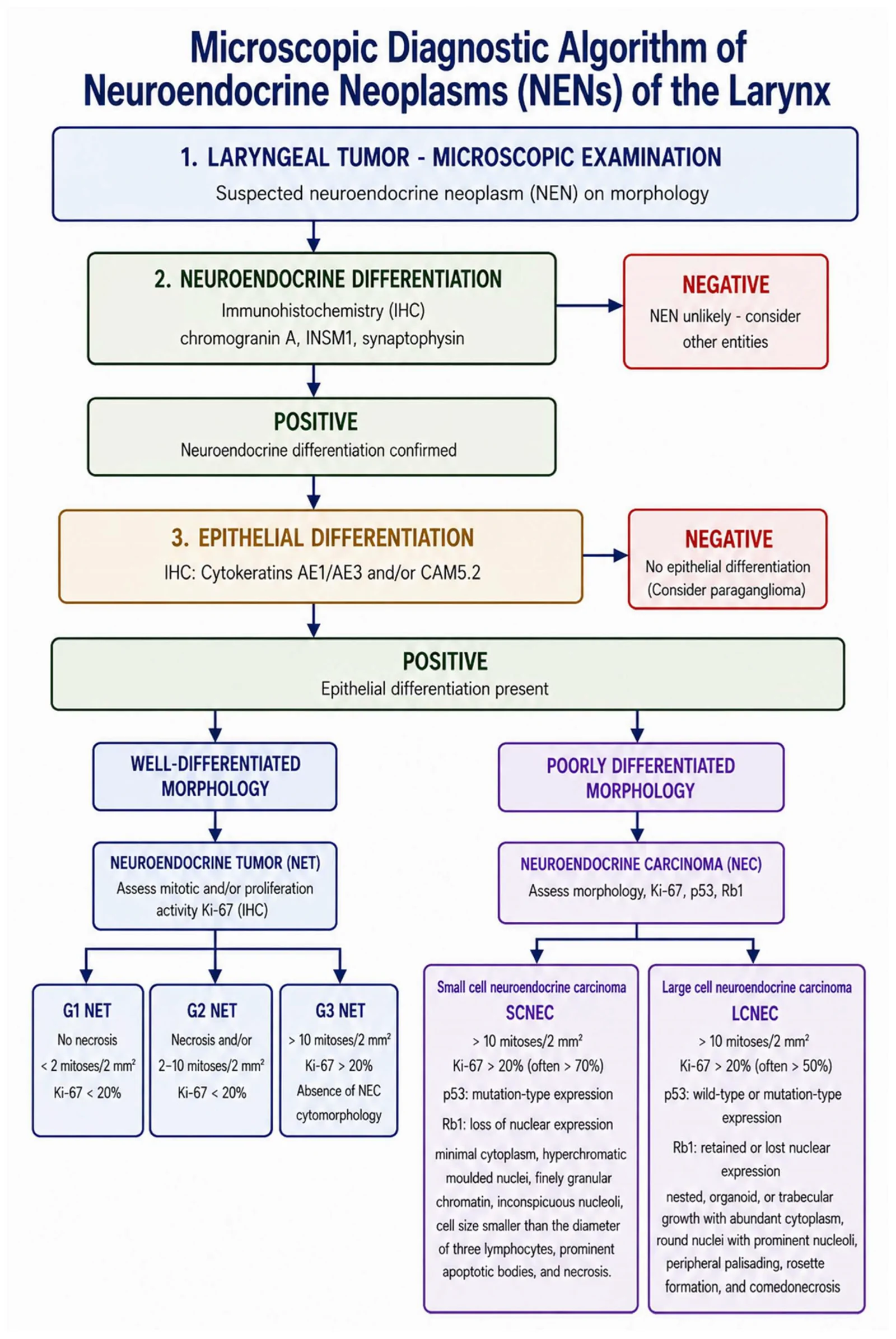
Microscopic diagnostic algorithm for laryngeal neuroendocrine neoplasms (NENs). The figure was created by the authors with the assistance of OpenAI ChatGPT (GPT-5.5) for graphical layout and design. The scientific content, diagnostic criteria, and final version were developed and verified by the authors.

## Data Availability

No new data were created or analyzed in this study. Data sharing is not applicable to this article.
